# Conceptualizing Automated Decision-Making in Organizational Contexts

**DOI:** 10.1007/s13347-024-00773-5

**Published:** 2024-07-16

**Authors:** Anna Katharina Boos

**Affiliations:** Department of Philosophy, University of Zurich, Zurich, Switzerland

**Keywords:** Algorithmic Decision Making (ADM), Artificial Intelligence (AI), Definition of ADM, Conceptual Analysis, Human-Computer Interaction (HCI), Socio-Technical Systems (STS)

## Abstract

Despite growing interest in automated (or algorithmic) decision-making (ADM), little work has been done to conceptually clarify the term. This article aims to tackle this issue by developing a conceptualization of ADM specifically tailored to organizational contexts. It has two main goals: (1) to meaningfully demarcate ADM from similar, yet distinct algorithm-supported practices; and (2) to draw internal distinctions such that different ADM types can be meaningfully distinguished. The proposed conceptualization builds on three arguments: First, ADM primarily refers to the automation of practical decisions (decisions to *φ*) as opposed to cognitive decisions (decisions that *p*). Second, rather than referring to algorithms as literally making decisions, ADM refers to the use of algorithms to solve decision problems at an organizational level. Third, since algorithmic tools by nature primarily settle cognitive decision problems, their classification as ADM depends on whether and to what extent an algorithmically generated output *p* has an action triggering effect—i.e., translates into a consequential action *φ*. The examination of precisely this *p*-*φ* relationship, allows us to pinpoint different ADM types (suggesting, offloading, superseding). Taking these three arguments into account, we arrive at the following definition: ADM refers to the practice of using algorithms to solve decision problems, where these algorithms can play a suggesting, offloading, or superseding role relative to humans, and decisions are defined as action triggering choices.

## Introduction

Public- and private-sector organizations are increasingly using software-based algorithms to support decision-making that directly affects individuals. This parctice is commonly referred to as “automated decision-making” or “algorithmic decision-making” (ADM). The areas of application are manifold, encompassing areas such as credit scoring, medical diagnostics, criminal justice, human resources, management, and advertising.^1^ The pervasive adoption of ADM and its potential for profound societal implications have catalyzed the emergence of a vivid interdisciplinary research community, devoted to examining the ethical challenges posed by this technology, including privacy violations (Becker, 2019; Wachter, 2020; Véliz, 2021), algorithmic discrimination (Barocas & Selbst, 2016; Chouldechova, 2017; Binns, 2022; Fazelpour et al., 2022; Lazar & Stone, 2023), and the lack of transparency and accountability (Lepri et al., 2018; Zerilli et al., 2019; Raji et al., 2020; Busuioc, 2020; Kroll, 2020).

Despite the increasing scholarly interest in ADM, little emphasis has been placed on the conceptual specification of the term (Lomborg et al., 2023). Gaining clarity on the conceptual foundations of ADM, however, can enrich our understanding of its societal implications. It provides a useful starting point for systematic investigations into the unique ethical challenges and social ramifications associated with ADM and how they vary across different types of ADM. Such insights are crucial for developing strategies to mitigate these challenges and to prevent unintended consequences (Ågerfalk et al., 2022). The aim of this article is therefore to elaborate a conceptualization of ADM that is tailored to organizational contexts and fulfills two main goals: (1) to demarcate ADM from similar, yet distinct algorithm-supported practices, and (2) to draw internal conceptual distinctions such that different subtypes of ADM can be meaningfully distinguished.

To my knowledge, the only study so far that has attempted to specify the concept of ADM is that of Richardson (2022), a legal scholar, who proposes the following definition for regulatory purposes:

“Automated Decision Systems” are any systems, software, or processes that use computation to aid or replace government decisions, judgments, and/or policy implementation that impact opportunities, access, liberties, rights, and/or safety. Automated Decision Systems can involve predicting, classifying, optimizing, identifying, and/or recommending. (Richardson, 2022, p. 795)

Although I remain agnostic about the usefulness of this definition for regulatory purposes, from a philosophical perspective, Richardson’s definition remains vague on two essential points. The first issue concerns the notion of decision-making entailed in ADM. According to Richardson’s definition, it is not merely “government decisions” that are subject to automation; but also “judgments” and “policy implementation.” It remains unclear, in what sense ADM precisely involves *decision-making* when applied to the latter two actions and, if the concept is uniformly applied to all three actions, what the notion of “decision-making” in ADM precisely means.^2^ The second issue with Richardson’s definition is that it also leaves open how we are to exactly understand the notion of “automation” in ADM. That is, to what extent and in what way must “computation” be involved in order for a decision process to be considered *automated*? In distinguishing between “aid” or “replace” decisions, Richardson, like most thinkers in the field, rightly observes that ADM systems vary in terms of how they are embedded in particular decision-making processes. However, she does not further discuss the criteria governing the attribution of a decision-making process to one or the other of these two categories. While Roehl (2022) and Sætra (2021) have proposed more useful typologies of ADM in this regard,^3^ they also leave room for more thorough conceptual groundwork about which features are crucial for the adoption of which specific ADM functions.

Thus, ADM remains a fuzzy concept, potentially causing misunderstandings as different authors make use of different conceptions. In order to tackle this issue and clarify the conception of ADM, the article develops three arguments. Section 2 focuses on the notion of “decision-making” entailed in ADM. I begin by arguing that, especially given the organizational context, ADM refers to the automation of *practical decisions* (i.e., decisions to *φ*) as opposed to *cognitive decisions* (i.e., decisions that *p*). Cognitive decisions come into play only insofar as they are *φ*-informing, and they thus play a subordinate role to practical decision-making. Second, I argue that it is misleading to conceive of ADM in terms of algorithms literally *deciding*, since, on the ontological level, algorithms lack the ability to choose and form intentions. Instead, the term ADM should be taken to refer to the use of algorithms *to solve* (practical) decision problems at an organizational level, where a “decision” is defined as an *action triggering choice*.

Section 3 sheds light on the meaning of “automated” in ADM. Although the notion of ADM refers to the automation of practical decisions, algorithms—taken in themselves—primarily settle cognitive decision problems. Therefore, the third argument contends that the qualification of a process as ADM ultimately depends on the extent to which an algorithmically generated output *p* has an action triggering effect—i.e., gives rise to a consequential action *φ*. This, in turn, depends on the institutional design of the organizational decision process. On the assumption that automation comes in degrees and with varying socio-technical configurations, it is precisely by looking at this *p-φ* relationship that we can pinpoint different types of ADM. On this basis, I will develop a typology that comprises four ideal types of algorithm-supported decision processes (preparing, suggesting, offloading, and superseding), of which only the latter three qualify as ADM. Hence, ADM is considered an umbrella term encompassing both semi- and fully automated decision processes, which are differentiated by the extent to which an algorithmically generated *p* determines the decisional output *φ*.

Finally, Section 4 synthesizes the three arguments and presents the following full definition of ADM as *the practice of using algorithms to solve decision problems, where these algorithms can play a suggesting, offloading, or superseding role relative to humans, and decisions are defined as action triggering choices.* The section closes with a discussion of how the proposed conceptualization meets the two goals set out at the beginning of this article and how it can contribute to future ADM research.

## Clarifying the Meaning of “Decision-Making” in ADM

### Cognitive and Practical Decision-Making

Kaufmann (1966, p. 25) distinguishes between two kinds of decisions. Decisions that determine that something is the case (i.e., decisions that *p*) are referred to as *cognitive decisions*—e.g., when a forensic psychiatrist decides, based on their assessment, that a defendant poses a limited risk of recidivism. In contrast, decisions that determine what to do (i.e., decisions to *φ*) are referred to as *practical decisions*—e.g., when a judge decides that the defendant should be paroled due to their favorable risk profile. It is the latter, practical notion of decision-making that is generally presupposed in decision theory (see Peterson, 2009). Moreover, it is possible to go a step further and argue that the term “cognitive decision” is a misnomer. Recognition theorists, for example, conceive of descriptive judgements “that something is the case” primarily in terms of *recognitional acts* rather than decisional acts, identifying them with the phenomenon of “recognition as *identification*” (Schaub, 2024, pp. 38–42; Ikaheimo and Latinen, 2007).^4^ Recognition as *identification* can involve identifying an object as having the property of being precisely this object (numerical), identifying an object as being an object exhibiting certain properties (qualitative), or identifying an object as being an object of a specific kind (generic) (Schaub, 2024, p. 40). Within this framework, it seems more appropriate to understand our forensic psychiatrist as qualitatively *identifying* the defendant as low-risk candidate rather than *deciding* that they are low-risk. Nonetheless, especially in cases where identification is essential but cannot be done with complete certainty, the use of the term “decision” does not seem entirely confused. While certain cases may be straightforward, our psychiatrist may encounter others with contradictory and inconclusive evidence, in which case they are ultimately forced to commit themselves in one direction or in the other. There is a point in time at which a decision must be made between the alternatives. However that may be, the semantic debate about the appropriateness of the term “cognitive decision” is ultimately less relevant to my argument then the effective distinction between the two phenomena—i.e., “determining that something is the case” and “determining what to do.” Therefore, for the sake of clarity and despite these terminological caveats, I will stick to Kaufman’s proposed terminology.

Since we are dealing with organizationally embedded ADM, our focus will primarily lie on practical decisions as opposed to cognitive decisions. Organizations are typically defined as “goal-directed, boundary-maintaining,^5^ activity systems” (Aldrich, 1979, p. 4) or, more specifically, “collectivities oriented to the pursuit of relatively specific goals and exhibiting relatively highly formalized social structures” (Scott & Davis, 2007, p. 29).^6^ Organizations identify themselves and maintain their raison d’être through their commitment to their organization-specific goal, which requires orchestrated action to achieve (Parsons, 1956; Barnard, 1971). Since action is inherent in the nature of organizations, so too are the decisions that shape these actions. Typically, the decisions organizations face revolve around the activities they undertake in pursuit of their organizational goals, thus being of a primarily practical nature. Organizations seldom engage in decision-making that does not, in some form, contribute to shaping future actions.

At the same time, there is not always a clear-cut distinction between cognitive and practical decision-making, and they are genreally not mutually exclusive. Cognitive decisions are likely to have a practical dimension as well, in the sense that once a belief or judgment is formed, it may impact future actions and manifest itself externally. In the same vein, practical decisions also have a cognitive dimension, in the sense that the choice of a particular course of action is likely to be preceded by a process of internal deliberation that has identified that particular action as the most suitable one. Decision-making often involves a series of lower-level decisions that must be settled along the way to solving the higher-level decision problem (Hansson, 2018). Typically, practical decisions to *φ* depend on cognitive decisions that *p*, while the *p* is supposed to provide the factual foundation for the decision in question. Notwithstanding the complexity and situatively emerging dynamics of real-life decision processes, I will generally assume here––for the sake of simplicity––that in organizational contexts, cognitive decisions are normally subordinated to practical decisions insofar as they play a *φ-informing* role.^7^

Against this backdrop, while organizations do engage in cognitive decision-making, they typically do so in order to solve practical decision problems, as in the example cited above of the forensic psychiatrist determining a defendant’s risk profile as a basis for the subsequent parole decision. There are plenty of other examples of this phenomenon: retail businesses estimating the expected demand for a specific product, to inform their stocking decisions; insurance companies defining which investments are considered sustainable to make wise investment decisions, environmental protection agencies assessing toxicity levels in water bodies to implement safety measures if needed, and so on. In all these examples, the reason why the organizations make cognitive decisions is that they are facing practical decision problems in the first place. This logic even applies to universities and research institutes, which, due to their role as knowledge producers, appear to hold a distinct status.^8^ However, their organizational goal is not just to generate knowledge, but also to disseminate and impart it in order to achieve added value for society, which invariably brings us back to the practical domain. Producing knowledge is thus an activity that goes beyond purely recognizing and establishing facts. Cognitive decisions, in the form of scientific findings that do not help immediately solve practical decision problems, are indeed part of the core business of research organizations. Nevertheless, these findings do not contribute to the research organizations’ goal unless they are made accessible to the research community and broader public. Scientific findings can only be recognized as such if practical decisions are made to disseminate them, be it in the form of publications, press releases, recommendations for action, educational offers, and alike. Consequently, even in the case of research organizations, cognitive decisions are not made purely for their own sake, but are rather tied to practical decisions pertaining to the fulfilment of their distinct role as knowledge producers.

Given the considerations outlined above, I will propose an account of organizational ADM that centers on to the automation of *practical* decision making rather than cognitive decision making. Cognitive decisions remain an important element in ADM, but only insofar as they inform practical decisions. The precise relationship between cognitive and practical decisions in ADM will be clarified in Section 4, where I will make the case that precisely this relationship is key to understanding the different degrees of automation involved in ADM. First, however, it is worth taking a closer look at the concept of decision-making entailed in ADM itself.

### The Standard Notion of a Decision as an “Intentional Choice”

For most theories of (practical) decision-making the notions of *choice* and *intention formation* are central elements (March, 1989, 1996; Chia, 1994). The element of choice is closely linked to the original meaning of “decision”: Etymologically, its roots are in Latin and can be linked to the term “incision,” which means “making a cut” (Chia, 1994, p. 795; based on Whitehead, 1925, p. 43). Thus, “decision” originally referred to cutting something off from a larger piece, which, in turn, presupposes that the larger piece is divisible, in the sense that it can be subdivided into individual, selectable items. It follows that a decision involves making a choice, which presupposes the availability of a range of alternatives or, at the very least, two options from which to choose. The term “option” here is understood as something, a particular entity, that can be selected from a range of possibilities, where non-selection can also constitute an option.

In what respect does the second element, intention formation, play a role in decision-making? According to Alfred Mele, “decision-making” is understood as a “momentary mental action of intention formation” (Mele, 2000, 2003, Chap. 9, 2022).^9^ To decide to *φ* means to actively form the intention to *φ*, whereas intentions are understood as “executive attitudes toward plans” (Mele, 2000, p. 100). When we make (practical) decisions, it is normally the case that we do not only *cognitively* identify option A as the best alternative available, but also actively form the intention *to do A*. We commit ourselves to an action plan, which is determined by the specific choice we make,^10^ meaning that decisions can be seen to “serve an executive function in relation to our subsequent action” (Pink, 1996, p. 66). On the standard conception, a (practical) “decision problem” is thus considered a problem whose solution involves the choice of one out of at least two action plans including a commitment to execute the selected action plan. A “decision” is, in turn, considered the final choice in a given decision problem for which the decision-maker has formed the intention to implement it.

The problem is that if decisions are defined in this way, the concept of ADM appears incoherent. There is an innate contradiction between the term “automated,” taken to mean “executed by algorithms,” and “decision-making,” understood in the sense of “intentional choice.” This contradiction is two-fold. The first issue is that when we say that someone “has a choice,” we normally mean that they are able to choose the option that they judge to be the most suitable based on their internal deliberation. Since the decision is theirs, the decisional output is determined at their discretion. In contrast, an algorithmic output is, by definition, the result of a predetermined set of computational instructions relating to a given input. The algorithm cannot choose to exercise discretion and decide otherwise. It receives an input, runs through the programmed calculation steps, and delivers the required result. For any given value *x*, the algorithm is determined in such a way as to give you the corresponding value *y*. Even in the case of machine learning (ML), where systems can determine decision criteria for a given problem without explicit human programming, the entire learning process is still algorithmically determined. As a result, algorithms do not choose what they judge to be the best option based on internal deliberation, but rather their choice is the result of a predetermined computational process.

The second issue is that if decisions are acts of “intention formation,” it implies that decision-makers are intentional agents. Intentions, like desires and beliefs, are propositional attitudes, meaning that they are mental states with representational content. The representational content of intentions is constituted by the corresponding action plan (Mele, 2000, p. 100). It is highly questionable, however, whether algorithmic systems have what is necessary to mentally represent propositional content and act according to it in a literal sense (Johnson, 2006). Although the behavior of these systems may be interpreted “as if” they had beliefs, desires, and intentions, they do not subjectively experience “what it’s like” (Nagel, 1974) to be in a particular mental state and do not genuinely understand its semantic content (Searle, 1980). On this basis, one might argue that the attribution of mental states to algorithmic systems can only ever be metaphorical, and thus that it seems ontologically misguided to say that algorithmic systems genuinely decide. Computers do not think; they calculate.^11^

### ADM as a Tool for Solving Decision Problems

If algorithmic systems are incapable of decision-making in this sense, does that mean that we should abandon the term ADM altogether? Not necessarily. One easy way out of this dilemma is, of course, to switch methodological lenses and adopt a functionalist approach, which would allow us to relax the ontological preconditions (see Dennett, 1987; Sullins, 2006; Laukyte, 2017; List, 2021). However, for those who believe that only beings with a mind can have genuine mental states—and thus by extension the ability to make decisions—this approach is quite unsatisfactory. Therefore, I propose to follow another route, one which does not require concessions regarding the ontological preconditions of a decision maker.

Given our focus on organizations, decision-making agency is to be localized at the organizational level. When officials, clerks, employees, and managers make decisions, they do so not as individuals making decisions about their personal affairs, but as representatives of occupational roles solving organizational problems. The choices they make in specific organizational contexts do not necessarily reflect their own personal preferences, nor do they necessarily form the intention to actually *φ* themselves. In a similar vein, instead of conceptualizing ADM in terms of algorithms genuinely *making decisions*, we can instead view ADM as a practice that *uses* algorithms to *solve decision problems* at an organizational level. This is the approach I will explore further here, discussing its implications for each element––choice and intention formation––separately.

#### The Notion of “Choice” in ADM

In order to further elucidate the notion of choice in the context of ADM, let us consider the following example: Suppose an educational institution is able to award five scholarships each year to exceptional students and, out of all the applications it receives, it must select the most promising ones. In ordinary language, we would—probably quite uncontroversially—call the final verdict a decision. Nevertheless, given the fact that the decision is embedded in bureaucratic processes, human discretion and, consequently, choice is likely to be diminished. Objective standards must be followed, which prevents bureaucrats from resolving decision problems based on their subjective perceptions. Their discretionary power is normally constrained by implicit and explicit rules, bureaucratic structures, and hierarchies that govern their roles. Decision-making power is generally attached to roles rather than individuals. For reasons of fairness and continuity, decision processes are often defined in such detail that it should (ideally) not matter who exactly reaches the final verdict.^12^ We can therefore say that the bureaucrat in our educational institution follows an algorithmic procedure to arrive at a decision in this matter.

Consequently, if we are inclined to say that ADM has nothing to do with decision-making because there is no “real choice,” then it would follow that a bureaucrat who rigidly applies predetermined selection criteria to settle a particular case is also not engaged in decision-making. By contrast, whenever bureaucrats have the liberty to make exceptions and exercise discretion, we can speak of a “decision problem.” Only in the latter case can the choice be considered a “real” one. Thus, the very same issue––such as the allocation of college grants––might be considered a decision in some cases but not in others, depending on the degree of standardization of the solution mechanism.

Pursuing this line of reasoning not only makes things unnecessarily complicated, but also misses the point: The method we choose to solve a given problem does not affect the nature of the problem itself. Suppose I am a very indecisive person and picking from the menu at a restaurant is usually a lengthy and cumbersome process, much to the annoyance of the hungry people I am with, who are all forced to wait patiently until I have made up my mind. Now suppose that in order to avoid dragging everyone down, I have developed an algorithmic procedure enabling me to quickly and easily make a selection from any menu. While I still have to decide what to eat each time I go to a restaurant, I now have a useful tool to facilitate this specific kind of recurring decision, namely *handing over* my choice to an algorithmic procedure. Even if this algorithmic procedure does not “decide” in the strong sense of the word (since it lacks the ability to make choices and form intentions), it does solve my decision problem.

Similarly, instead of handling every grant application individually, the relevant agency can, in order to facilitate the process and ensure equal treatment of applicants, put in place a standardized procedure. As in the scenario outlined above, the actual decision problem is *handed over* to a fixed procedure, carried out by a human official. ADM simply takes this a step further, replacing the human with a computer program. Seen in this way, ADM is not substantially different; it just further restricts human discretion.

Consequently, the notion of “choice” entailed in decision-making derives from the fact that the problem (which the decision aims to settle) involves picking one out of at least two options. This notion of choice does not depend on the procedure by which an option is ultimately selected. It does not matter whether the choice is made by weighing all options against each other in an internal process of discretionary deliberation, or by applying a standardized mechanism in which the final verdict is already predetermined. In other words, the fact that a choice is made does not depend on whether or not the chosen problem-solving strategy involves discretion. One might say that it is up to the decision-maker how much (situational) discretion they want to allow for a particular class of decision problems in any given instance.

To see how this translates when applied to ADM, consider one of the simplest cases, namely speeding enforcement, which has been automated for quite some time (Bovens & Zouridis, 2002, pp. 179–180; Delaney et al., 2005). If the speed measured exceeds the limit, the driver whose license plate is captured automatically receives a fine. At first sight, it might seem counter-intuitive to even label this a “decision,” given that a simple predetermined trigger-response mechanism governs the issuing of the fine. If a car exceeds the speed limit, this automatically triggers a reaction; if it does not, nothing happens. On closer inspection, however, the picture looks different. Suppose the speed camera is connected to an algorithmic system placed in a residential area with a speed limit of 30 km/h. The following process takes place: The camera measures the speed of each passing car. The speed value *x* is then assessed by an algorithm using the simple equation *y* = 30 – *x*. If *x* is greater than 30, then *y* is negative; if it does not, then it is greater or equal zero. In order to determine whether to issue a fine or not, the algorithm applies the simple rule “if *y* ≥ 0, then NO_FINE, if *y <* 0, then FINE.” The resulting output will then trigger further actions leading to the issuance of a fine.

As the situation presents itself, the process cannot be completed until the algorithm has selected one of the two available actions for each car (where NO_FINE also counts as an action here). This means that for each passing car, the system *settles a decision problem*, which consists of selecting one of the two possible courses of action. Clearly, the speed enforcement agency could include more variables in the equation, so that the decision does not merely concern a fixed speed limit but involves a complex construct comprising many situation-specific factors that are considered simultaneously (e.g., traffic load, weather, road condition, individual vigilance, etc.). The agency could even decide that there will be no fixed threshold, leaving it up to a human official or a sophisticated AI system to evaluate whether a speed *x* is considered adequate at a specific moment in time *t* and under the specific conditions *c*. Nonetheless, I see no conclusive argument to support the claim that the latter, more complex solution mechanism should be considered more of a decision problem than the former, simpler solution mechanism which focuses on a fixed threshold value. Ultimately, both boils down to the very same decision problem: choosing between the two options FINE or NO_FINE. The fact that the agency opted for a more sophisticated resolution mechanism in the latter case, allowing a greater discretionary leeway, does not change the nature of the problem. From this perspective, even a simple rule-based automated procedure with a single variable qualifies as ADM, provided that the decision problem is solved by means of the selection of one out of at least two alternative courses of action. The upshot is that our notion of decision making in the context of ADM does neither depend on discretion nor complexity.

#### The Notion of “Intention” in ADM

Now that we have clarified the meaning of choice in relation to ADM, we can turn to the question of intention formation. Continuing to use the same approach, we can conceptualize ADM as the practice of using algorithms to solve decision problems at an organizational level. If we frame the problem in this way, intention formation plays out at the level of the organization and not at that of the algorithmic system—provided we follow the standard conception of decisions as intentional choices. This obviously presupposes that organizations qua group agents are capable of forming intentions, a presupposition I will accept in line with the prevailing position in the academic literature.^13^ Providing a comprehensive ontological account of how to properly understand intention formation at the organization level falls outside the scope of this paper. In any case, it is not immediately relevant to the argument here. Instead, let us focus on the reason *why* the conceptual element of intention formation in decision-making seems necessary in the first place. The reason is that intention formation is precisely what distinguishes practical decision-making from cognitive decision-making. Unlike cognitive decisions, practical decisions may include, but are not limited to, theoretical judgments about a specific matter. We also settle on an action plan and make a resolution to execute it.^14^ To exemplify, in practical decisions we do not merely decide which menu item is most appealing (which taken for itself would qualify as cognitive decision), but which menu item we are going to order based on what we find most appealing. Therefore, whenever a practical decision is made, it implies that, at that moment in time, the choice is made with the attitude that it will be actually implemented.

This action-committing attitude is crucial for understanding the difference between practical decision-making and cognitive decision-making and since the proposed account of ADM relies on it, we need a proper concept to capture it. Within this context, however, the choice of terminology ––be it “intention” or some other term––does not meaningfully affect the argument here. For the sake of simplicity, I therefore propose referring to the notion of “*action triggering* to capture this attitude and define (practical) decisions as “*action triggering* choices” as opposed to “intentional choices.” This effectively captures the essence of practical decisions in contrast to cognitive decisions, at least to the extend as needed for our conceptualization of ADM, while simultaneously sidestepping profound ontological discussions on intention formation at an organizational level. As in the case of “intentional choice,” the notion of “*action triggering* choice” implies that we can reasonably expect this choice to entail a concrete action plan that ultimately triggers the implementation of this very choice—provided, of course, that the decision-maker acts rationally and sticks to their plan (which, at least in the context of formal organizations, we are normatively inclined to do).

What does “action triggering” mean exactly in the context of ADM and how can it help to identify ADM cases? Since we are interested in identifying whether some decision processes qualify as ADM, whereas others do not, it is of little use to retrospectively determine, on a case-to-case basis, whether or not individual decisions had action triggering effects. Instead, we need to be able to identify ADM ex-ante and assess whether the process is, in principle, such that it qualifies as ADM. After all, organizations can, for a variety of reasons, fail to act as intended and end up *not* triggering the planned consequential actions. Therefore, we need to assess the action triggering element of a decision in terms of whether a decision can contingently come into action once the relevant choice has been taken. Given that we are looking at an organizational context, the triggering of consequential actions is only guaranteed if it is attached to clear institutionalized roles that are automatically activated once the relevant choice has been taken. Along these lines, the triggering condition can be operationalized via the *existence of an institutional implementation mechanism* which ensures that the respective consequential action for a given choice is being triggered. This allows us to assess in principle whether the institutional setting in an organization is such that, were the decision to be made, it would have action triggering effects. As a result, the classification of ADM depends to a considerable extent on its institutional setting.

By way of illustration, suppose a company’s marketing department uses an intelligent Customer Relations Management (CRM) program. It has many features, some of which are used on a daily basis and some of whose potential is far from fully exploited. In the latter case, the program is equipped with sophisticated data-mining capacities that are able to create fine-grained customer profiles, classifying them into different target groups. In this way, or so the original idea runs, the company can better tailor their marketing activities to its audience. However, it turns out that, as a result of limited resources, insufficient procedural formalization, or simply bad management, no one in the company is or feels responsible for actually implementing the consequential actions (i.e., targeted marketing measures). There is no role in the company charged with ensuring that the respective measures targeting the different groups are being taken. Even if the program theoretically advocates further action, we cannot consider its outputs “practical decisions” as they are not attached to institutional implementation mechanisms that effectively trigger the corresponding actions. Conversely, if the procedure were such that the output initiated a cascade of further actions whose function was to implement the choice (i.e., marketing measures according to respective target group classification), then the program would be operating in the domain of practical decision-making, thus qualifying as ADM.

To conclude this section, let me take stock of the argument so far: I have clarified the notion of “decision-making” in the context of ADM, characterizing it as making *action triggering choices* for decision problems that occur at an organizational level. Both components are considered necessary: Whereas the notion of *choice* presupposes the availability of at least two options concerning the further course of action, the notion of *action triggering* presupposes the institutionalization of the organizational procedures necessary for the choice to be implemented in principle.

Having defined in this way the meaning of “decision-making” in ADM, we can now turn to clarifying the meaning of “automated”: What does it mean to say that, in ADM, algorithms “are used to solve decision problems”? To what extent must algorithms be involved in the decision-making process for the term ADM to be applicable? The remainder of the article will focus on addressing these questions.

## Clarifying the Meaning of “Automated” in ADM

The notion of “automation” generally refers to any system “operating or acting, or self-regulating, independently, without human intervention” (Nof, 2009, p. 14). In the context of ADM, “automated” usually refers to the use of algorithmic systems to solve decision problems with limited human input. These systems vary in technical sophistication, ranging from rule-based systems (where algorithms implement predefined “if then that” rules written by human programmers) to ML*-*based systems (where algorithms apply complex mathematical functions, allowing a system to “learn” from data without explicit human programming). Regardless of their mathematical complexity, however, when we think about automation, the question is to what extent these tools operate without human intervention. For this reason, it is necessary to examine the precise role that algorithms play relative to humans in the decision-making process. Importantly, since ADM does not refer to the automation of any task but specifically *decisions* in the sense outlined above, the mere fact of employing algorithms to facilitate or implement decisions is not sufficient for the process to be classified as ADM. The crucial point is that the *act* of making a decision is to be *automated*, meaning that the final choice in a given decision problem is determined by the algorithm. Put differently, it is the extent to which an algorithm determines the decisional output without direct human involvement that makes a decision automated.

To illustrate this point, let us recall the example of the college grant we discussed earlier. If the grantees were to be selected by an algorithm, the case would clearly qualify as ADM. Consider, however, the following variation on this scenario: It is still the administrator working in the education office who decides which candidates are to receive the scholarship. However, to do so, they rely on a piece of software, let us call it “BestStudent,” which stores all the applications and makes them accessible in a structured way along with some preliminary analysis: After scanning all the application documents, it generates a spreadsheet in which the performances of all the candidates are listed in a way that makes them easy to compare. Furthermore, the program also leverages this information by predicting the likelihood that each applicant will complete their degree and graduate on time (henceforth “degree completion outcome”) (see Demeter et al., 2022). Let us further assume that “BestStudent” is connected to another program, call it “PayStudent,” which is employed by the education office to automatically process the payment transactions for grantees. Once the official has made their final choice, the rest of the process will be handled automatically via “PayStudent.” While the decision in question is both *prepared* and *implemented* by algorithms, the process does not qualify as ADM. Granted, “BestStudent” significantly facilitates the decision process but it is still on the administrator to actively select the candidates and, if necessary, make trade-offs. Decision-making is thus *prepared*, but not determined by algorithm. Similarly, even though the final verdict is *implemented* automatically, this process does not involve any further decisions as “PayStudent” merely executes the instructions it receives.

We may not always face cases that are so clear-cut, in the sense that the act of decision-making is free from automation, while both the preparation and implementation involve automation. Moreover, in our highly digitized society, the chances of finding organizational decision processes that involve no technology whatsoever are small. Likewise, even in the technologically most advanced organizations, we rarely encounter processes that are entirely detached from human involvement. After all, technology is embedded in social institutions. ADM is therefore best conceived of as a hybrid process in which humans and algorithms closely interact in the decision-making process and where automation is a matter of degree and various socio-technical configurations (Bader & Kaiser, 2019; Selbst et al., 2019; Peeters, 2020; Roehl, 2022).

This raises the question of where to draw the line: Where does decision *automation* start? Several attempts to classify ADM have led to the identification of ideal typical subtypes (Alter, 1977; Bovens & Zouridis, 2002; Sætra, 2021; Roehl, 2022). These typologies generally follow a similar logic, with the decisive feature being the extent to which the algorithm impacts the final decision. The subtypes are usually arrayed along a spectrum representing gradually diminishing human discretion and gradually increasing automation. Most subtypes in the various proposed typologies can be classified into one of the three categories: non-automation (humans determine the decisional output with only limited technological support), semi-automation (humans and technology jointly determine the decisional output), and full automation (technology determines the decisional output without direct human involvement).

All these attempted classifications implicitly share a practical notion of decision-making. As mentioned earlier, given that our focus is on organizational ADM, this delimitation is reasonable and should be emphasized: ADM, as conceptualized here, pertains exclusively to the automation of practical decisions. This is not only because organizational decision-making is, generally speaking, primarily practical (notwithstanding a few exceptions), but also because this delimitation avoids conceptual under-specificity. If we included the automation of mere cognitive decisions in the concept of ADM, then almost any result arrived at with the help of computational tools could be considered ADM: Otherwise, almost any result arrived at with the help of computational tools would be considered an automated decision: the solution of an equation determined by a pocket calculator, the p-value fixed by a statistical program, the output of an automatic text translation program, the precipitation probability predicted by the weather forecast, a CCTV camera identifying a shape as a person, and so on. I therefore suggest that unless these tools are embedded in a process for solving practical decision problems at the organizational level, they do not qualify as ADM.

Upon closer inspection, however, a problem emerges: No algorithmic tool does anything other than compute. They solve questions such as “What is the value of *Y* given the underlying model and a particular value of *X*?” or “What is the probability of a given data point *x*_*i*_ belonging to a particular class label?” In solving these mathematical problems, algorithms can be seen as determining facts, and hence, making choices about theoretical matters—e.g., “among all the conceivable values of *Y*, value *y* is considered the most accurate answer” or “given the three class labels *A*, *B*, *C*, datapoint *x*_*i*_ most likely belongs to *A*.” When examined in isolation, algorithmic tools at best qualify as *cognitive* decision tools, making decisions “that *p*.” To enter the practical domain, and thus the domain of ADM as defined here, their output *p* must have action triggering effects and play a decisive role in determining a consequential *φ*. This, in turn, depends on the institutional mechanisms established in the organization. Consequently, if we put the pieces together, ADM comprises a process which, in one way or the other, translates algorithmic *cognitive* decisions into *practical* decisions. The crucial question is how.

To answer this question, it is worth examining in more detail how cognitive and practical decisions relate to each other in ADM. That is, how does the algorithmically generated *p* eventually affects the consequential *φ*. Analyzing this relationship will put us in a better position to systematically pinpoint the various socio-technical configurations and degrees of automation that are conceivable in ADM. Inspired by existing ADM classification studies (especially Sætra, 2021, and Roehl, 2022), I identify four ideal types of algorithm-supported decision-making that vary in terms of how the *p* translates into the *φ*. These are: (1) preparing, (2) suggesting, (3) offloading, and (4) superseding. These four ideal types are elaborated on below. Although the typology proposed here does not fundamentally conflict with the aforementioned classifications, it aims to further strengthen the conceptual foundations of ADM by providing a more fine-grained understanding of the functional differences between various ADM types.

### Ideal Type (1): Preparing

Ideal type (1) refers to algorithmic tools that, as illustrated above, play a *preparing* role in relation to human decision-makers, as they provide support in rendering relevant information accessible and offering valuable data insights. In doing so, they contribute to preparing the factual foundations on the basis of which human decision makers can make well-informed decisions. A variety of use cases can be subsumed under this category, ranging from tools to retrieve, present, and analyze descriptive data (Alter, 1977) to tools to boost data intelligence via AI-powered analytics. As such, these tools do *not* determine the decisional output (i.e., the consequential action *φ*), and they thus fall outside the domain of ADM. Instead, they perform one of two functions: The first function is to support human decision-makers in determining the *p* that eventually grounds the decision to *φ*. Consider the example of “BestStudent:” It is structured in such a way that the decision about whom to award special college grants to (the decision to *φ*) requires a determination about who among the applicants is deemed particularly promising (decision that *p*). “BestStudent” facilitates the determination of this *p* in two relevant ways: On the one hand descriptively, by structuring the data in such a way that the administrator can more easily determine the *p*, and on the other hand inferentially, by assigning each candidate a predictive score concerning the degree completion outcome, thus providing the administrator with an additional variable to take into account when determining the *p*, i.e., who is among the most promising applicants. Crucially, however, this predictive score, let us call it *p*_*a*_, does not constitute the complete *p*, let us call it *p*_*d*_, which is needed for the final decision to *φ*, i.e., ﻿whom to award the college grants to. It is just one of numerous variables feeding into the official’s deliberation process, such as the previous school performance, the persuasiveness of the motivation letter, or the personal impression made during the interview. Thus, the first function of ideal type (1) corresponds to algorithms that support human decision-makers in determining a *p* (for the sake of informing a *φ*) by presenting, analyzing, synthesizing, and inferring relevant information. The algorithmically generated output *p*_*a*_ feeds into the determination of the final *p*_*d*_ (informing the decision to *φ*), but leaves it essentially incomplete. Thus, *p*_*a*_*≠ p*_*d*_.

In contrast, the second function of ideal type (1) systems corresponds to algorithms that are capable to generate a complete *p*, but leave the corresponding *φ* essentially incomplete. Thus, *p*_*a*_ *= p*_*d*_, but the corresponding *φ* is not implied on the procedural level, that is, the decision problem is not yet structured in such a way that is already clear which specific *φ* should be taken in response to a given *p*.^15^ The decision-maker retains the discretion to decide what to do with the algorithmically provided information and to select the most suitable consequential action *φ* on this basis. Consider the case of an environmental and civil defense bureau that uses sophisticated software to model and forecast potentially disastrous events, such as earthquakes, avalanches, floods, and so on. There are few empirical values that can be relied upon to determine at which threshold which specific safety measure should be taken, meaning that the relationship between the *p* and the consequential *φ* has not yet been systematically established. Imagine a situation in which an algorithm predicts a medium to strong earthquake in a specific region. It is on this factual basis that a seismologist must assess the situation and decide on the next steps (e.g., whether to give safety instructions to potentially affected citizens, protect critical infrastructure, evacuate particularly vulnerable residents, etc.). While the algorithm provides essential information about the expected situation, it does not draw any conclusions about potential further actions.

In summary, algorithms in ideal type (1) play a *preparing* role relative to humans as they provide valuable insights on the basis of which humans can make well-informed decisions. Nevertheless they only provide the factual basis for a decision and do not indicate which actions should follow. This is still up to humans. The relevant *p*-*φ* relationship is too loose to qualify as ADM, either because the algorithmically generated output *p*_*a*_ is not sufficient to constitute the complete *p*_*d*_ on which the consequential *φ* is eventually based (*p*_*a ≠*_*p*_*d*_) or, if it does (*p*_*a =*_*p*_*d*_), because it is still unclear which *φ* is to follow from the given algorithmic output *p*. Hence, we face a *machine-out-of-loop*-situation,^16^ in which the algorithmic output does not significantly affect the consequential action *φ*. This, in turn, means that humans still play a decisive role in determining the decisional outcome (Sætra, 2021, p. 33). Consequently, ideal type (1) falls into the category of *non-automated decision-making*.

### Ideal Type (2): Suggest

In ideal type (2), algorithms play a *suggesting* role relative to human decision-makers by recommending concrete courses of action (Alter, 1977, p. 42; Roehl, 2022, p. 48). In contrast to (1), the algorithmically generated output is not only sufficient to constitute the *φ-*informing *p* (thus, *p*_*a =*_*p*_*d*_), but it also implies on the procedural level which specific *φ* is to follow from which specific *p.* The algorithm can, in principle, access all the necessary decision variables needed to determine the corresponding *φ*. There is already an institutionally established link between the algorithmically generated *p* and the consequential *φ*. Nevertheless, the defining feature of *suggesting* algorithms is that they are limited to issuing recommendations: For any given *p*, they automatically determine a provisional consequential action (*φ*_*prov*_), which will then be reviewed by decision-makers who have the final word in determining which actual consequential action will be triggered (*φ*_*trig*_). The “BestStudent” algorithm would fall into this category if it took into account *all* the relevant variables for the final decision and presented a complete list of students who were classified as most promising. In this case the algorithmic output (i.e., the list) would not represent an explicit recommendation of action (i.e., “award candidates x, y, z”), as in the speeding example (“FINE” or “No_FINE”). Still, it fulfills virtually the same function and little needs to be done to transform it into a recommendation for action. Especially in highly structured decision problems, the link between the *p* and the *φ*_*prov*_ is so clearly established that the *φ*_*prov*_ is already implied in a given *p*. Hence, the algorithmic output in (2) may contain either an explicit recommendation for action or a value of *p* that clearly implies the recommended consequential action to human decision-makers.

In the *suggesting* ideal type of ADM there is still a *human-in-the-loop* who has the discretion to choose whether or not to approve the suggested decision (*φ*_*prov*_). Thus, human input is needed to translate *φ*_*prov*_ into *φ*_*trig*_. If the algorithmically suggested *φ*_*prov*_ is approved, it turns into *φ*_*trig*_ and hence will be triggered (*φ*_*prov*_ = *φ*_*trig*_). If *φ*_*prov*_ is rejected, it is possible for humans to override the *φ*_*prov*_ and instead select a different action *φ*_*trig*_ to be triggered (*φ*_*prov*_ ≠ *φ*_*trig*_). This process may not only involve re-examining the factors considered by the algorithm and understanding how precisely the algorithm arrived at this conclusion (provided the algorithm is transparent) but can also include consulting additional sources to gain a more thorough insight of the case. In this latter scenario, *suggesting* algorithms would come closer to functioning as *preparing* algorithms as in ideal type (1). The decisive difference, however, is that for ideal type (2), consulting additional information is *not necessarily* required to make a decision, whereas for ideal type (1), it is.^17^ Note that the classification does not depend on whether the consequential action that is actually triggered is executed by humans or machines. What matters is who, human or machine, ultimately determines the *φ*_*trig*_, not how the *φ*_*trig*_ is triggered or implemented.

Furthermore, as empirical studies show, there may be discrepancies between a system’s intended design and its actual use.^18^ It is the actual human-machine interactions that are decisive for identifying its ideal type. Suppose we have an algorithmic system that is designed to generate *suggested* decision results, yet the *human-in-the-loop* completely ignores them, always overriding them and deciding entirely based on their gut feeling. Even if the system would technically qualify as ideal type (2), in reality it would not even qualify as (1). Conversely, imagine a scenario in which the human decision-maker blindly trusts the algorithmic suggestion and accepts it without questioning. Under these circumstances, there is hardly any difference with respect to cases of full automation (see ideal type (4) as discussed in § 3.4).

### Ideal Type (3): Offloading

In ideal type (3), the relation between the *p* and the consequential *φ* is even more clearly established than in the previous ideal type. It is structured in a similar way to (2), except that the algorithmic system automatically executes the suggested course of action *unless* a human vetoes it. That is, the *φ*_*prov*_ automatically translates into *φ*_*trig*_ unless there is human intervention (*φ*_*prov*_ = *φ*_*trig*_). Ideal type (3) algorithms thus play an *offloading* role in relation to human decision-makers by automatically processing cases deemed unproblematic, thereby reducing their decision load and freeing up resources for decisions that specifically require human expertise (see Sætra, 2021, p. 34).^19^ Besides software agents, ADM systems of this type may also include embodied artificial agents such as robots and autonomous drones that are integrated into organizational structures. They typically perform actions and decisions autonomously, but still remain subject to human oversight and intervention when necessary.

Our “BestStudent” example would qualify as ideal type (3), if the system, starting from the list of algorithmically selected grantees, automatically proceeded to the next step of issuing the payments, unless a human intervened. As a different example, consider the fraud detection tools used by insurance companies to flag seemingly suspicious policyholders (Eubanks, 2018). Based on these red flags, policyholders are subjected to increased scrutiny and as long as this process is pending, the benefits are withheld. In the context of *offloading* ADM, it is the algorithm that determines which individuals are scrutinized. The algorithmic output is not a suggestion, but an *instruction* that initiates the review process, with all that it implies. Yet in order to prevent false accusations and imposing an unjustified burden on mistakenly flagged individuals, insurance officers can still intervene and re-evaluate putative borderline cases.

As in the case of ideal type (2), we are dealing with hybrid decision processes, even if algorithms play a more significant role in determining the decisional output. Yet these cases still belong to the domain of *semi-automation*, with a *human-on-the-loop* situation. In contrast to human-*in*-the-loop situations, where humans are still decisive components in the decision loop (because they actively translate the *φ*_*prov*_ into *φ*_*trig*_), in human-*on*-the-loop-situations the loop is seamless, unless a human actively intervenes. The line between type (2) and (3) systems is admittedly blurry, as can be seen in the example of fraud detection. A case worker’s judgment that a particular algorithmic instruction does *not* seem odd could be interpreted as tacit approval, which would move the case into the domain of (2). Yet, there still seems to be a categorical difference between actively approving and actively denying the translation from *φ*_*prov*_ to *φ*_*trig*_. When it comes to assigning use cases to the ideal types, what matters is the attitude humans maintain toward the algorithmic outputs and the practices they adopt in handling them. A human-*in*-the-loop situation is different from a human-*on*-the-loop situation, insofar as humans keep an eye on *every* algorithmic output before approving it, which is different to situations in which humans only keep an eye on outputs that are obviously dubious. Moreover, note that type (3) systems often focus on processing relatively clear-cut cases, referring the more complex and ambiguous ones to human case workers, who then take a decision based on the provided information (see, e.g., Ranerup & Svensson, 2023). In that case, ADM systems that generally serve an *offloading* function also exhibit elements of type (1) *preparing* systems, yet they handle the majority of cases without the need for human review.

### Ideal Type (4): Superseding

Ideal type (4) refers to cases in which decision processes are entirely delegated to algorithmic systems. This means that we are dealing with cases in which algorithmic tools are *superseding* humans in their role as decision-makers in one of two senses: (a) they are taking over roles and decisions that were previously performed by humans or (b) they are taking on new roles and decisions that could not have been previously performed by humans.^20^ Decisions in ideal type (4) therefore feature the most explicit *p*-*φ* relationship. They are structured, insofar as for any algorithmically generated *p*, there is a consequential *φ* already inscribed in the process that is automatically triggered. In other words, the *φ*_*prov*_ is automatically translated to *φ*_*trig*_. There is no room for humans to intervene in the process and re-evaluate the decisional output in real time.^21^ Our automated speed limit enforcement example fits nicely into this category: Once speeding is detected, the driver automatically receives a fine. “BestStudent” would similarily fall into this category if the system handled the determination of college grant recipients without any direct human involvement or potential intervention. We could also think of a more complex ADM system, such as a software robot employed by a consumer product company to monitor stock levels and order new goods based on calculations of expected demand. The robot autonomously decides when and how much of each good needs to be ordered and executes the associated actions without human input.

Consequently, *superseding* ADM corresponds to decision processes that keep humans *out-of-the-loop* and thus are *fully automated.* Recall that it is irrelevant whether the actual triggering or implementation of the determined *φ*_*trig*_ is executed by humans or machines. In any case, *superseding* ADM does not necessarily mean that humans are not involved in any capacity, it is simply that humans are not involved to the extent that they could directly affect the decisional output. Their role is limited to works preparing the data for the system to operate, performing maintenance work, implementing the tasks assigned by the ADM system, monitoring system performance and reporting functional issues, making sure the system is aligned with its intended objectives, and so on.

The table below captures all four ideal types at a glance. Even if ideal type (1), by definition, falls outside the scope of ADM, it is still worth including it in the typology to illustrate where decision automation begins, thus drawing a line between ADM and non-ADM (Table 1).

**Table 1:**
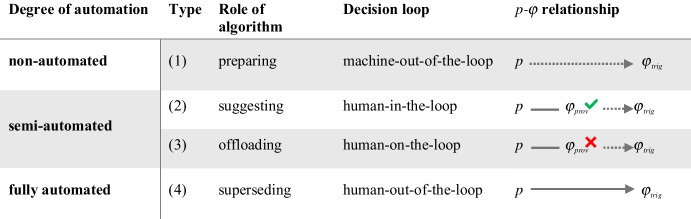
Ideal types capturing various levels of automation in algorithm-supported decision-making

The typology builds on existing classificatory work for algorithmic decision support and automation systems, most notably Sætra (2021) and Roehl (2022). However, it goes beyond mere descriptions of a tool’s functional role relative to human decision-makers and adds a further layer systematizing how exactly an algorithmic output translates into the actual decisional output, and how differences in this respect result in different functional roles. This close-up view makes it possible to pinpoint more precisely what conceptually determines the different functional roles of algorithms in decision processes. On this basis, we can now see more clearly what differentiates non-automated decision-making from semi- or fully automated decision-making: For a decision to count as *(semi-)automated*, the selected action *φ* must be procedurally implied by the algorithmically generated *p*. This, in turn, depends on two conditions: First, algorithms must generate a complete *p* constituting the decisive factual foundation for the decision in question (*p*_*a*_ = *p*_*d*_) and, second, this *p* must immediately translate into a corresponding *φ* that is being triggered (either with or without human review).

The typology defines ideal types and thus remains inherently incomplete with respect to the diverse manifestations that ADM can assume in practice. Firstly, since the deliniation of the different ideal types is rather fluid, some ADM systems might fall in between categories––e.g., when in type (2) all suggestions are being accepted without questioning, except for those that are clearly off. In that case, there is no significant diffence to (3). Secondly, a system’s design may deliberatiely incorporate elements from various ideal types––e.g., when systems are a combination of (1) and (3), in a way that they automatically execute the clear cut cases and prepare the more complex ones for human processing. Thirdly, actual ADM usage may deviate from a system’s originally intended design, while this deviation may even vary across the different users of the very same system––e.g., when in type (2) individual case workers adpot different attitudes towards the system, thus assigning different weights to algorithmic suggestions compared to their colleagues. Notwithstanding these empirical challenges, the aim of this typology is to systematize the different automation levels in ADM, based on the extent to which algorithms settle decision problems without direct human involvement. The fact that some real-world cases remain ambiguous, does not undermine the validity of the concptual distinctions drawn by the typology.

Moreover, by capturing automation in a purely functional manner, the typology remains agnostic to both, the complexity of the underlying decision problems and the complexity of the algorithmic models in use. The latter two are related insofar as the complexity of a decision has consequences on how automation can be technically implemented. 22 Automating more complex decision problems requires more complex algorithmic models compared to simpler ones, and the complexity of a decision can limit the possibilities for automation.^23^ However, while complexity may affect the potential for automation, once it is technically feasible, it does not necessarily affect the degree of automation. The degree to which an algorithm independently determines a decisional output does not depend on model complexity. Whether a decision problem is solved by assessing a single, fixed threshold parameter or by evaluating multiple parameters using complex mathematical equations, is irrelevant for the question of whether and to what extent the problem is solved by algorithm. Therefore, the typology accommodates a broad spectrum of algorithmic models with varying degrees of technical sophistication across all levels of automation.

However, the advent of the new AI generation has sparked great optimism that unprecedented possibilities are being unlocked. With the availability of more advanced algorithmic models, the automation of decisions previously deemed too complex for algorithmic processing may now be in reach. More importantly, advanced AI models not only broaden the potential scope of ADM, but promise to enhance the decision quality based on actionable insights from accurate predictions, nuanced simulations, and previously hidden data patterns. In that case, decision complexity is deliberately raised with the aim of obtaining better results. The use and proliferation of ADM systems may therefore be increasingly driven by their dual ability to both automate and *augment* decision-making processes. In contrast to automation, the essence of *augmentation* does not necessarily lie in replacing humans, but in enhancing their capabilities with AI, allowing for a richer and deeper understanding of specific issues (Veale & Brass, 2019; Benbya et al., 2021; Jain et al., 2021). We can thus think of decision *augmentation* as enhancing the cognitive dimension of decision-making (thus pertaining to the *φ*-informing *p's*), which pays off by creating a more robust evidence base that eventually leads to more effective practical decisions.

Notwithstanding the significance of this development, it does not affect the construction of our typology as presented here. The augmentative capacity of an ADM system which typically correlates with its technical sophistication and model complexity, does, as argued above, not impact the system’s degree of automation, and vice versa. In principle, each of the ideal types (1)–(4) can exhibit varying degrees of augmentation while maintaining a constant of automation, and different degrees of automation are conceivable at the very same level of augmentation. In this respect, the typology proposed here neither follows Sætra (2021), who conceptualizes augmentation via the construction of distinct ideal types along the spectrum of automation, nor Roehl (2022), for whom the difference between rule-based and more sophisticated ML-based systems is decisive for the degree of automation. To fully account for different degrees of technical sophistication and their corresponding augmentative potentials, we should integrate an additional dimension into our so far one-dimensional typology––one that exists independently from automation. This, however, would be a subject for a different paper. For the time being, focusing exclusively on automation, our typology accounts for a wide range of algorithmic models with varying degrees of technical sophistication and associated augmentative capacities.

## Conclusion

Taking into account what has been discussed so far, we can provide the following definition:

*ADM is the practice of using algorithms to solve decision problems, where these algorithms can play a suggesting, offloading, or superseding role relative to humans, and decisions are defined as action triggering choices.*

Given our focus on organizational contexts, ADM primarily refers to the automation of *practical decisions* (i.e., decisions to *φ*) as opposed to *cognitive decisions* (i.e., decisions that *p*). Cognitive decisions are still involved in ADM, but only insofar as they play a *φ*-informing and thus subordinate role to practical decision-making.

At the outset of this article, I formulated two goals, namely that my proposed conception should: (1) meaningfully demarcate ADM from similar, yet distinct algorithm-supported practices; and (2) draw internal distinctions such that different subtypes of ADM can be meaningfully distinguished. Regarding (1), classifying a decision process as ADM depends, first, on whether the algorithm-supported process actually qualifies as a *decision*, which necessitates: (i) the selection of one out of at least two possible courses of action *(choice)* and (ii) the immediate triggering of a consequential action *φ* resulting from the preceding choice (*action triggering).* Second, this decision must qualify as automated, which depends on whether the algorithms involved play a sufficiently decisive role in determining the decisional output. Since, when taken in themselves, algorithmic tools are primarily cognitive decision tools, their algorithmically generated *p* must translate into a consequential action *φ*, as part of the decision-making process. This, in turn, depends on the relevant institutional setting. Therefore, the decisive criterion for a decision process to qualify as *(semi-)automated* is whether (iii) the selected action *φ* is already procedurally implied by the algorithmically generated *p*, meaning that the algorithmically generated *p* immediately translates into a corresponding action *φ* that is being triggered (either with or without human review). Criteria (i)–(iii) equip us with the necessary conceptual tools to successfully classify ADM and demarcate it from related practices, such as automated processes that are not considered decisions or decisions that—although algorithmically supported—are not considered automated.

With regard to goal (2), I started from the assumption that automation comes in degrees and in a variety of socio-technical configurations. I argue that these variations can be pinpointed precisely by looking at the *p*-*φ* relationship in the decision process. I developed a typology capturing four ideal types of algorithm-supported decision processes, only three of which qualify as ADM. As the decisive feature is the extent to which the algorithmically generated *p* determines the decisional output *φ*, the ideal types line up on a spectrum of gradually diminishing human discretion over the decisional output and thus an increasing degree of automation. Hence, ADM is conceptualized as an umbrella term that encompasses both *semi-automated* decision processes (represented by the ideal types (2) *suggesting* and (3) *offloading*) and *fully automated* decision processes (represented by ideal type (4) *superseding*). Ideal type *(1) preparing* does not qualify as ADM, since the link between the algorithmically generated *p* and the consequential action *φ* is not sufficiently established. The algorithmic output remains inconclusive with respect to either the *p* needed to determine the consequential *φ* or the specific *φ* which is to follow a given *p.* In contrast to existing classifications, the typology proposed here can capture more systematically and in a more nuanced way how exactly the subtypes differ with regard to automation.

In light of recent efforts to provide ethical guidance to the practice of ADM, it is crucial to arrive at a clear understanding of the applications that fall within its scope. Moreover, the proposed typology serves as a starting point for systematically assessing the different implications for each different ADM type, with a particular focus on their level of automation. In this context, special attention is to be drawn on how ADM systems operate in their context specific environment, as it might reveal major discrepancies between the intended design of a system and its actual use. Some case workers might just blindly follow an algorithmic suggestion, whereas others will only follow an algorithmic instruction when they have independently reached the same conclusion. The proposed typology provides an anchor to identify these discrepancies.

However, the typology does not come without limitations. Being agnostic to the complexity of the underlying decision problems, the proposed conception of ADM encompasses a wide range of algorithmic models with varying degrees of technical sophistication and associated augmentative potentials. These are not captured by the typology, as the degree of automation in ADM does not depend on technical sophistication. However, technical sophistication can have other relevant implications. It is widely recognized that as technical sophistication increases, so too does the opacity of the underlying algorithmic models. This, in turn, may pose major challenges to the explicability, accountability, and ultimately the legitimacy of ADM. Systematizing different ADM types in terms of their level of opacity could therefore be greatly beneficial for advancing future research on ADM and its ethical implications. As for this article, however, I hope the present analysis has made its own contribution to strengthening the conceptual foundations of ADM and that it will stimulate further research.

## Data Availability

Not applicable.

## Footnotes

1 For a general overview of the proliferation and use ADM in Europe see AlgorithmWatch, 2019, 2020.

2 A similar lack of clarity is also found in other contributions, which in the course of their normative assessment of ADM provide brief definitions mainly focused on the algorithmic nature of ADM, but do not elaborate further on the key notion of a “decision” (see Zarsky, 2016; Lepri et al., 2018; Oswald, 2018; Selbst & Barocas, 2018; Zweig et al., 2018; Cobbe, 2019; Scantamburlo et al., 2019, Zerilli et al., 2019, Shrestha et al., 2019; Grote & Berens, 2020; Mökander et al., 2021).

3 Note that Sætra (2021) does not directly address the term “ADM” in proposing his “Typology of AI Applications in Politics.” However, his analysis is specifically tailored to decision-making with or by AI in politics, which situates his typology in the realm of ADM.

4 Recognition as *identification* is, along with recognition as *acknowledgement* and recognition as *interpersonal recognition*, considered one of three phenomena in which the concept of recognition manifests itself (Schaub, 2024, pp. 38–42; Ikaheimo and Latinen, 2007).

5 In the sense that they maintain a clear distinction between members and non-members.

6 Note that this definition specifically applies to “formal organizations,” a term which typically refers to highly professionalized (mostly legal) entities that are characterized by a high degree of bureaucratization (see Blau, 1957; Scott & Davis, 2007; Weber, 2008). It is mainly in this environment that ADM is being increasingly adopted, as opposed to more informal and less institutionalized collectivities.

7 This obviously carries with it the normative assumption that organizations are (or at least seek to be) rational decision-makers, implying that they (ideally) make well-informed decisions serving their best interest (given the information available at the time) (see Savage, 1954; Simon, 1955; March, 1996).

8 I am grateful to an anonymous reviewer for critically drawing my attention to this point.

9 Similar notions of decision-making are found in Kaufman, 1966, p. 34; McCann, 1986, p. 254; Pink, 1996, p. 3; Clarke, 2003, Chaps. 1 & 7.

10 On the notion of commitment in relation to intentions, see also Bratman, 1987, Chap. 2.

11 Such line of argument is typically defended by philosophers who hold that intentionality is grounded in phenomenal consciousness (also known as the “phenomenal intentionality thesis”), which in turn supervenes on intrinsic properties of the mind (see Searle, 1980, 1984, 1994; Johnson, 2006; Horgan, 2013). For general accounts defending the phenomenal intentionality thesis, see Siewert, 1998; Kriegel, 2003; Horgan et al., 2004; Graham et al., 2017; Mendelovici, 2018. In contrast, adherents of the so-called “instrumentalist approach”—which is influentially represented by Dennett’s “intentional systems theory” (Dennett, 1987)—do not essentialize mentality, and thus do not make any presumptions about the ontological make-up of mental states. According to Dennett, we are dealing with an intentional system insofar as the behavior of that system, be it human, animal, or machine, can be meaningfully interpreted and predicted by adopting the “intentional stance,” which involves treating the system “as if” it were a rational agent guiding its actions based on its “beliefs” and “desires.” Instrumentalist theories should be understood in the broader context of functionalism, a position in the philosophy of mind that defends the view that mental sates are constituted by their functional roles rather than their specific internal structure (Kobes, 2009; Levin, 2021). On this view, there is no reason of why intentional agency—and thus, in principle, the concept of decision-making—cannot be genuinely attributed to artificial entities. Prominent philosophers who adopt a functionalist-instrumentalist stance towards artificial agents include Sullins, 2006; Laukyte, 2017; List, 2021.

12 This is especially true in the context of public-sector organizations representing state authority. However, since we are dealing with organizations that are highly institutionalized and, as such, exhibit a clear division of roles, governed by rules and hierarchies, the analysis also holds to a great extent for private-sector organizations.

13 One could again voice skepticism here and call into question the view that organizations qua group agents have intentions and thus can genuinely make decisions. This is because organizations as such lack phenomenal consciousness, and thus do not have minds and mental states. (For a defense of group-consciousness see Schwitzgebel, 2015; see List, 2018, for a contrasting position.) Nevertheless, even if one denies the possibility of groups having literal “minds” and being subjects in “their own right” (see, e.g., Pettit, 2003; List & Pettit, 2011; Tollefsen, 2015), a non-metaphorical account of organization-level intentions is still plausible: Organizations can be said to have intentions because their members have intentions and these individual intentions are related to each other (see Tuomela, 1989, 2007; Bratman, 1993, 2021; Ludwig, 2014, 2017). For authors who are skeptical of group agency and thus of the existence of organization-level intentions, see Velasquez, 1983, 2003; Szigeti, 2014; Rönnegard, 2015.

14 We may say, however, that when making *cognitive* decisions (i.e., when deciding that *p*), we form the belief that *p*, and along with it, the intention to stick to that belief. However, once we form that intention we are no longer just engaged in a cognitive decision. This is because our plan to stick to our belief that *p* ultimately concerns our future behavior and thus our actions, which brings us back into the realm of practical decision-making. I would therefore defend the view that for purely cognitive decisions, we do not form intentions (understood as executive attitudes towards action plans).

15 Distinguishing between *p*_*a*_ and *p*_*d*_ is no longer necessary here, since the algorithmically generated *p*_*a*_ equals the complete *p*_*d*_, informing the decision to *φ*.

16 The idea of capturing human-machine interactions via the concept of so-called “decision-loop-constellations” originates from the debate on “killing robots” in military ethics, where one distinguishes between human-in-the-loop weapons, human-on-the-loop weapons, and human-out-of-the-loop weapons (Citron & Pasquale, 2014, pp. 6–7). Citron & Pasquale (ibid.) were the first to transfer this concept to the field of algorithmic scoring (i.e., automated predictions) and helped it gain popularity in the context of ADM. See, e.g., Danaher (2016) and Sætra (2021).

17 I am grateful to an anonymized reviewer for pushing me to elaborate more clearly on the distinction between ideal type (1) and ideal type (2).

18 As Lomborg et al., 2023 emphasize, ADM systems are uniquely embedded in complex socio-political, economic, and professional contexts, which can quite differently influence the norms and incentives for how case workers interact with these systems. In some cases, case workers might develop work arounds to bypass system rigidity (Dolata et al., 2020), thereby increasing their discretion compared to what the system’s design originally intended. In other cases the discretionary scope might be reduced compared to what was initially intended, either because case workers might gradually increase their confidence in the system over time, not seeing a need to reassess the algorithmic output (Ranerup & Svensson, 2023), or because they lack the necessary professional skills to effectively scrutinize the system’s suggestions (Saxena & Guha, 2024). Therefore, ADM systems always have to be examined within their specific contextual environment.

19 The logic behind *offloading* ADM-systems is similar to Robotic Process Automation (RPA) that is increasingly introduced in the public sector (Juell-Skielse et al., 2022a). RPA systems are software robots that emulate human workers performing tasks in the same user interfaces as their human co-workers (Berg, 2022). They usually take over repetitive administrative tasks to alleviate the burden on human workers but are also increasingly capable of taking over more complex tasks due to AI (Juell-Skielse et al., 2022b). However, since the concept of RPA refers to the automation of tasks in general (such as, e.g., updating data records) and not necessarily to the automation of *organizational decisions*, they should not be seen as a specific example for ideal type (3). In fact, RPA solutions can be found across all ADM types. If they are used to automate the provision of relevant information for making decisions, they fall under ideal type (1). If they automate the generation of a suggested decision result, the system falls under ideal type (2), and if this suggestion is automatically executed unless vetoed by a human, the RPA falls under ideal type (3). Finally, if the RPA comprises the automation of the entire decision-making process, it falls under ideal type (4). I am grateful to an anonymous reviewer for highlighting the close relationship between ADM and RPA.

20 Note, however, that in ideal types (1)–(3), too, algorithmic tools enable new forms of preparation, suggestion, and offload that were previously unconceivable for humans. This is especially true of AI applications that surpass humans in their cognitive abilities.

21 This does not, of course, mean that decisions cannot be retrospectively appealed and declared invalid.

22 I am grateful to an anonymous reviewer for stressing this point.

23 Note that, as discussed in § 2.3, decision complexity primarily refers to the solving mechanism, not the nature of the problem itself. The very same problem (e.g., choosing between A or B) can be solved by applying various mechanisms with different degrees of complexity.
